# Sensitivity to change of the Beach Questionnaire to behaviour, attitudes and knowledge related to sun exposure: quasi-experimental before-after study

**DOI:** 10.1186/s12889-015-1415-0

**Published:** 2015-01-31

**Authors:** Teresa Fernández-Morano, Magdalena de Troya-Martín, Francisco Rivas-Ruiz, Nuria Blázquez-Sánchez, Agustín Buendía-Eisman

**Affiliations:** Doctoral student at the University of Granada, Granada, Spain`; Dermatology Department, Costa del Sol Hospital, Marbella, Spain; Red de Investigación en Servicios de Salud en Enfermedades Crónicas–Redissec, Granada, Spain; Research Department Costa del Sol Hospital, Marbella, Spain; School of Medicine, Granada University, Granada, Spain

**Keywords:** Health questionnaire, Validation, Sensitivity to change, Sun exposure habits, Attitudes to sun, Knowledge about sun exposure

## Abstract

**Background:**

Health questionnaires must present accredited measurement properties such as validity, reliability and sensitivity to change, the latter being essential for interventions to be planned and for evaluating their effectiveness. The aim of this study was to evaluate the sensitivity to change of a Beach Questionnaire.

**Methods:**

Quasi-experimental before-after study carried out in 2011, for a study population of adolescents attending schools in the Costa del Sol. First, the questionnaire was administered to the adolescents, after which a multicomponent educational intervention was carried out; finally, three months later, the same questionnaire was re-administered to the same adolescents. Changes were assessed in the categories of each item, using the McNemar test, and the changes in the scores, standardised to a range of 0–100, using the Student *t* test for paired samples, and including the mean of the differences and the 95% confidence interval. The level of statistical significance was set at p < 0.05.

**Results:**

228 adolescents, aged 14–17 years, and 55.3% were girls. Statistically significant changes were observed in sunburn experiences, exposure to the sun at mid-day and attitudes to sun exposure and suncreams. For the seven items related to knowledge about sun exposure, a higher rate of correct answers was observed. The analysis of changes, within the standardised range, revealed a significant improvement in the scores for sun exposure habits (MD 4.33; CI 95% 2.2-6.5), attitudes to sun exposure (MD 2.22; CI 95% 1.2-3.2) and knowledge (MD 9.10; CI 95% 7.1-11.1), but not in those for sun-protection practices (MD 0.23; CI 95% -1.2-1.7).

**Conclusions:**

The Beach Questionnaire on behaviour, attitudes and knowledge related to sun exposure is the first such instrument in Spanish language to provide sufficient sensitivity to change. It constitutes a useful tool for epidemiologic research into photoprotection and for skin cancer prevention programmes.

## Background

The application and analysis of health questionnaires is the most widely used instrument in the study of sun exposure behaviour. The planning and development of interventions, the evaluation of their effects, and numerous epidemiological studies are based on the data obtained from such questionnaires. Nevertheless, on many occasions only limited time and effort are invested in their design [1,2]. To be effective, questionnaires should have accredited measurement properties, that is, validity (the capability to measure the concept for which they were designed), reliability (the capability to measure without error) and sensitivity to change (the capability to detect changes). This latter quality is essential if the questionnaire results are to be properly used to evaluate the effectiveness of an intervention [3]. In recent decades, several countries have conducted skin cancer prevention campaigns and educational interventions in schools aimed at improving attitudes and behaviour regarding solar protection and at reinforcing knowledge in this respect [4-6]. To determine the real impact of such interventions, appropriate measuring instruments must be employed and their psychometric properties correctly evaluated.

In 2009, our research group published its first questionnaire on behaviour, attitudes and knowledge related to sun exposure, a questionnaire that was validated in its Spanish language version. The corresponding study of this questionnaire confirmed the validity and reliability of the scale used [7]. The present study aims to assess the sensitivity to changes in questionnaire responses, for use in future interventions in the field of skin cancer prevention.

## Methods

### Design

Quasi-experimental before-after study focused on evaluating sensitivity to changes in responses to the Questionnaire.

The study was conducted in three phases in 2011: 1) pre-intervention phase: the questionnaire was administered from January to March (the responses given at that time referred to the previous year, 2010); 2) a multicomponent educational intervention was carried out in May and June; 3) a post-intervention phase, in which the same questionnaire was administered to the same adolescents three months after completing the intervention (the responses given referred to the summer of 2011).

### Scope

Western Costa del Sol (southern Spain) is a mainly tourist-oriented and residential area with a permanent population of 379,334 inhabitants [8], although the floating population exceeds one million inhabitants in the summer months. Sunbathing on the beach is one of the main attractions for Spanish and foreign tourists in this area.

Population and sample: 200 adolescents aged 14 to 18 years were recruited from secondary schools. To obtain a population size of over 200 individuals completing the questionnaire, pre-and post-intervention, a loss rate of 30% was assumed and an average class size of 25 students was estimated. Therefore, the initial sample size assumed was 300 subjects. Among the 42 secondary schools in the area, 12 classes were selected from simple random sampling of the total, using EPIDAT 3.1 software.

All research was conducted in strict compliance with the Declaration of Helsinki. The ethics committee of the Costa del Sol Hospital has declared that this study protocol is exempted from the requirement for a statement of ethical approval, due to the public health intervention nature of the study. The exemption was obtained before the study began.

To administer the questionnaire, an initial request for participation in the study was addressed to the directors and tutors of each of the classes in the participating schools; subsequently, the parents or guardians of the participating adolescents were informed and their consent was sought. In these classes, randomly selected, all students were invited to participate in the study. Adolescents who did not speak Spanish were excluded from the study population, as the questionnaire is validated exclusively for a Spanish-speaking population [7].

All the data compiled in this project were recorded anonymously, in strict accordance with the laws and rules on data protection currently in force under Spanish legislation (Act 41/2002 of 14 November; Act 15/1999 of 15 December).

### Questionnaire

The questionnaire was developed by a group of experts on the basis of studies of sun-related behaviour, and taking into account questionnaires examined in both English and Spanish-language studies. Subsequently, the psychometric properties of validity and reliability were tested, in the latter case with respect both to internal homogeneity and to test-retest stability. The validity of the concept was evaluated by an exploratory principal components factor analysis, from which we obtained two domains for the block of 6 items concerning protection practices, accounting for 55% of the variance, and 4 domains for the block of 14 items concerning attitudes, which accounted for 64% of the variance. The internal consistency of the items for each of the resulting factors was examined using Cronbach’s alpha coefficient, taking significant alpha values as those above 0.70. The stability of the items was estimated by test-retest, assessing the proportion of absolute agreement and intraclass correlation coefficient, for all sections except that of knowledge, for which the delta statistic was used; all items met these criteria [7].

The questionnaire contained the following sections: 1) Demographic data (3 items); 2) Colour of unexposed skin (1 item); 3) Fitzpatrick phototype (1 item); 4) Habits of sun exposure at the beach (3 items, number of days and number of hours/day [5 response categories] and number of hours/day in the middle of the day [4 response categories]; 5) Sunburn experiences on the beach (1 item), with five response categories (none, 1–2, 3–5, 6–10 or more); 6) Sunscreen practices at the beach (6 items, Likert response format in 5 categories - never, rarely, sometimes, usually, always); 7) Attitudes related to the sun (14 items, 5-category Likert response format, from totally disagree to totally agree); 8) Knowledge related to sun exposure (7 items) with dichotomous response (true or false).

To compare domains with different numbers of items, the following scales were standardised in the range 0–100 [9,10]: habits of sun exposure, sun protection practices, attitudes regarding the sun and knowledge related to sun exposure. Higher scores indicated low sun exposure, good sunscreen practices, positive attitudes to sun protection and good knowledge of sun exposure.

### Intervention

Multicomponent educational interventions of proven efficacy [11-14] were selected for analysis and adapted to the Spanish context, taking into account the results obtained from our prior baseline study [15]. A multi-component intervention was conducted during school hours, with a duration of about 90 minutes and delivered by a dermatologist with experience in occupational training. During this intervention, a slide show provided information about sun exposure, its benefits and risks (such as skin cancer and photoaging), and protection measures, transmitting a positive message. Next, a workshop was held to examine sun-protection creams, the teenagers were familiarised with a dermatoscope [16] and with the ABCD rule for the early detection of melanoma, and educational videos were shown. At the end of the session, questions were answered and the adolescents contributed ideas or comments on photoprotection. As an additional motivating activity, each class prepared a collage setting out positive and negative messages on photoprotection.

### Statistical analysis

A descriptive analysis was performed of the individuals who completed the questionnaire pre and post-intervention, with measures of central tendency and dispersion for the quantitative variables and of frequency distribution for the qualitative ones. Knowledge of the question was determined by reference to the number of correct answers given. For sun exposure habits, protection practices, attitudes and knowledge, changes were assessed in the categories of each item by reference to the results for the pre and post-intervention questionnaires, using the McNemar test, and the changes in the scores, standardised to a range of 0–100, using the Student *t* test for paired samples, including the mean of the differences and the 95% confidence interval. In all the analyses, the level of statistical significance was set at p < 0.05.

## Results

In the first phase of the study, 308 students were initially recruited, from eleven different schools, eight of which were public and three, private. In this first phase, 38 students were excluded because informed consent was not obtained. Thus, the pre-intervention was completed by 270 students, representing 87.6% of the initial group. After the educational intervention at the participating schools, the post-intervention questionnaire was completed by 228 students, i.e., 84.4% of the pre-intervention sample and 74% of the initial sample.

Among the population of 228 adolescents on which this study was based, 55.3% were girls, 46.9% were aged 14 years and 86.8% were Spanish. 54.3% had light or very light skin and 77.6% were phototype III or IV (Table 1).

**Table 1:** **Descriptive data of the sample of adolescents who completed the test-retest**

**Variables**	**n**	**%**
**Sex**		
Male	102	44.7
Female	126	55.3
**Age**		
14	107	46.9
15	43	18.9
16	59	25.9
17	19	8.3
**Nationality**		
Spanish	198	86.8
Foreign	30	13.2
**Skin colour**		
Very light	14	6.1
Light	110	48.2
Light brown	62	27.2
Brown	42	18.4
**Phototype**		
I	11	4.8
II	40	17.5
III	97	42.5
IV	80	35.1

In the analysis of change in sun-exposure habits after the intervention, statistically significant differences were observed in the number of hours the study subjects remained on the beach at mid-day (p = 0.049), and borderline significant differences were observed for cases of sunburn (p = 0.051).

Among the sun-protection practices considered, analysis revealed changes in the use of sunshades (p = 0.006) and in avoiding exposure on the beach at mid-day (borderline significant, p = 0.052) (Table 2).

**Table 2:** **Test-retest change in habits of sun exposure and photoprotection practices**

**Item**	**N**	**Change +**		**No change**		**Change -**		**p**
		**n**	**%**	**n**	**%**	**n**	**%**	
Habit: days’ sunbathing on the beach	228	73	32.0	112	49.1	43	18.9	0.108
Habit: hours per day	224	75	33.5	101	45.1	48	21.4	0.068
Habit: mid-day sun exposure	222	73	32.9	100	45.0	49	22.1	0.049
Sunburn experienced during the previous summer	227	60	26.4	131	57.7	36	15.9	0.051
Practice: sunshade	225	44	19.6	99	44.0	82	36.4	0.006
Practice: sunglasses	227	62	27.3	101	44.5	64	28.2	0.167
Practice: sunhat	215	45	20.9	118	54.9	52	24.2	0.529
Practice: long-sleeves, long trousers	221	11	5.0	193	87.3	17	7.7	0.690
Practice: avoid mid-day exposure	225	78	34.7	97	43.1	50	22.2	0.052
Practice: use suncream	227	69	30.4	102	44.9	56	24.7	0.547

Analysis of pre vs post-intervention change showed that in three of the 14 items in the questionnaire section on attitudes to sun exposure, there were statistically significant differences for the attitudes “I like the feeling of the sun when I’m lying on the beach” (p = 0.002), “I like to sunbathe” (p = 0.001) and “It’s worth using sun-protection creams in the future” (p = 0.025) while the change in the attitude “When I go to the beach I’m more comfortable in the shade” was of borderline statistical significance (p = 0.056) (Table 3).

**Table 3:** **Test-retest change in attitudes related to sun exposure**

**Item**	**N**	**Change to more agreement**		**No change**		**Change to less agreement**		**p**
		**n**	**%**	**n**	**%**	**n**	**%**	
Attitude: When I am brown, my clothes suit me better	228	44	19.3	127	55.7	57	25.0	0.599
Attitude: Sunbathing helps prevent health problems	227	63	27.8	93	41.0	71	31.3	0.282
Attitude: I like the feeling of the sun when I’m lying on the beach	226	36	15.9	112	49.6	78	34.5	0.002
Attitude: It’s worth using sun-protection creams in the future	227	62	27.3	136	59.9	29	12.8	0.025
Attitude: I find sun-protection creams unpleasant	225	67	29.8	90	40.0	68	30.2	0.533
Attitude: It’s worth using sun-protection creams even if I don’t get a tan	227	77	33.9	97	42.7	53	23.3	0.226
Attitude: People who are tanned are more attractive	227	56	24.7	119	52.4	52	22.9	0.844
Attitude: Sunbathing is good for my body	228	60	26.3	98	43.0	70	30.7	0.195
Attitude: Sunbathing relaxes me	227	41	18.1	113	49.8	73	32.2	0.097
Attitude: Being tanned makes me look young and relaxed	228	60	26.3	104	45.6	64	28.1	0.297
Attitude: Sunbathing cheers me up	227	46	20.3	116	51.1	65	28.6	0.136
Attitude: I like sunbathing	227	32	14.1	123	54.2	72	31.7	0.001
Attitude: When I go to the beach I’m more comfortable in the shade	227	82	36.1	99	43.6	46	20.3	0.056
Attitude: I don’t like high-protection sunblockers because they are unattractive	228	53	23.2	101	44.3	74	32.5	0.133

For the seven items related to knowledge about sun exposure, a higher rate of correct answers was observed in the post-intervention assessment, with statistically significant differences (p < 0.001) for the following items: 1) “If I use sunblock cream I can sunbathe without risk” (68.7% pre-intervention and 92.1% post-intervention); 2) “Avoiding sun exposure at an early age decreases the risk of skin cancer by 80%” (51.6% vs. 70.2%); 3) “Once my skin is brown, I don’t need to continue using sunscreen” (87.7% vs. 96.9%) (Table 4).

**Table 4:** **Test-retest change in knowledge related to sun exposure**

**Item**	**N**	**Pre-test success rate**		**Post-test success rate**		**p**
		**n**	**%**	**n**	**%**	
1: Sun protection creams prevent the aging of the skin caused by the sun’s rays	226	174	77.0	185	81.9	0.20
2: The sun is the main cause of skin cancer	226	213	94.2	219	96.9	0.18
3: The sun causes skin blemishes	225	219	97.3	221	98.2	0.73
4: If I use sunblock cream I can sunbathe without risk	227	156	68.7	209	92.1	<0.001
5: Avoiding the sun at the hours of peak intensity (12.00-5.00 pm) is the most effective way to protect the skin from the sun	225	197	87.6	205	91.1	0.20
6: Avoiding sun exposure at an early age (under 18) decreases the risk of skin cancer by 80%	225	116	51.6	158	70.2	<0.001
7: Once my skin is brown, I don’t need to continue using sunscreen	227	199	87.7	220	96.9	<0.001

The analysis of changes in the scores, standardised to a range of 0–100, revealed a significant improvement in the scores for three of the four sections: sun exposure habits, attitudes to sun exposure and knowledge about sun exposure (p < 0.001) (Table 5).

**Table 5:** **Changes in the scores, standardised to the range 0–100, for the pre and post-intervention questionnaire sections**

**Section**	**N**	**Pre-test (range 0–100)**		**Post-test (range 0–100)**		**p**	**Mean difference**	**Confidence interval 95%**	
		**Mean**	**SD**	**Mean**	**SD**			**Lower**	**Upper**
Sun exposure habits	219	30.4	18.4	34.7	18.2	<0.001	4.33	2.2	6.5
Sun protection practices	204	29.4	12.5	29.7	12.8	0.76	0.23	−1.2	1.7
Attitudes toward sun exposure	216	57.4	11.2	59.6	10.9	<0.001	2.22	1.2	3.2
Knowledge	223	80.4	15.8	89.5	12.0	<0.001	9.10	7.1	11.1

## Discussion

This study, of an educational intervention aimed at adolescents, highlights the sensitivity to change of the Beach Questionnaire, designed to assess behaviour related to sun exposure. Sensitivity to change is defined as the ability of the measuring instrument to detect changes when an intervention or treatment of known efficacy is performed [17].

In this study, a multi-component intervention based on previously-evaluated models was addressed at a target group of adolescents. Previous studies have concluded that this age group presents high risk behaviour and is most susceptible to persuasion to change [11-14]. Analysis of the pre and post-intervention results revealed changes had taken place in the answers given to different components of the questionnaire (habits of sun exposure, sunburn experience, sun protection practices, attitudes and knowledge). These changes were statistically significant for some items, with sensitivity being greatest for the questions related to sun exposure at mid-day (sun exposure habit and sun protection practice), sunburn episodes in the most recent summer, attitudes to sun and suncreams (“I like the feeling of the sun on my skin”, “I like sunbathing”, “It’s worth using sun protection creams”) and knowledge (“If I use sunblock cream I can sunbathe without risk”, “Avoiding sun exposure at an early age decreases the risk of skin cancer”, “Once my skin is brown, I don’t need to continue using sunscreen”). By contrast, the items related to photoprotection practices (the use of suncreams and long-sleeved clothes) presented low sensitivity values; this was also true for certain attitudes on suntans and some aspects of knowledge. Perhaps changes in these aspects can only be achieved by maintaining long-term behavioural interventions. With respect to the students’ knowledge of these questions, the results obtained can be attributed to the ceiling effect evident in some of the items, because the baseline level of knowledge was already good. The changes observed are not only consistent but also significant, as they resulted from just one educational intervention, performed over a short period of time.

Although questionnaires have been shown to be valid instruments for measuring sun-protection behaviour [18], they present considerable variability and comparison is not easy, whether in a single country, between different countries or between different age groups, and so the development of standardised questionnaires represents a great improvement [19,20].

The questionnaire analysis performed in this study will facilitate future epidemiological studies of conduct related to recreational sun exposure, which is the major risk factor for the development of skin cancer; moreover, it contributes to the planning and evaluation of interventions on photoprotection in the context of skin cancer prevention programmes, which is an area of special interest for application in schools.

Of recent studies that have assessed questionnaires regarding sun exposure, some present individual measurement properties that are valid and reliable [1,19,21-24] but to date none have described the sensitivity to change. Therefore, the present study describes the first questionnaire on habits, attitudes and knowledge related to sun exposure at the beach, with sufficient validity, reliability [7] and sensitivity to change, for use in the field of epidemiologic research and health promotion.

Among its limitations, this study may be affected by selection bias; however, the adolescents included in the study population attend various schools, both public and private, in the Costa del Sol area. Moreover, the measures analysed may be limited by memory errors by the subjects, by the difficulty of estimating the frequency of habits and by social influence; moreover, there have been a differential memory bias with respect to the previous summer in the subject’s responses to the first questionnaire (in the winter) and the second one (after the summer). Nevertheless, the approach adopted is probably the most common means of evaluating this and other areas of health care, such as diet, physical activity and smoking. Another possible limitation is the fact that the intervention took place over a relatively short period of time; even so, the questionnaire is sensitive to changes in some of the sections. In the future, we intend to assess the interventions that produced the greatest impact. Finally, it should be noted that the questionnaire refers to sun exposure at the beach; this environment could usefully be extended to include other areas when sun exposure takes place, such as pools, parks and other recreational areas.

## Conclusions

In conclusion, we have developed the first questionnaire on behaviour, attitudes and knowledge related to sun exposure to provide sufficient validity, reliability and sensitivity to change. This questionnaire constitutes a useful tool for epidemiologic research and for skin cancer prevention programmes, as well as for evaluating educational interventions to implement prevention campaigns in Spain and other Spanish-speaking populations. Its use in other countries will require studies of transcultural adaptation and its validation for other languages, to ensure linguistic equivalence and similar psychometric properties in the new cultural environment [25].
